# Quantized anomalous Hall resistivity achieved in molecular beam epitaxy-grown MnBi_2_Te_4_ thin films

**DOI:** 10.1093/nsr/nwad189

**Published:** 2023-07-04

**Authors:** Yunhe Bai, Yuanzhao Li, Jianli Luan, Ruixuan Liu, Wenyu Song, Yang Chen, Peng-Fei Ji, Qinghua Zhang, Fanqi Meng, Bingbing Tong, Lin Li, Yuying Jiang, Zongwei Gao, Lin Gu, Jinsong Zhang, Yayu Wang, Qi-Kun Xue, Ke He, Yang Feng, Xiao Feng

**Affiliations:** State Key Laboratory of Low Dimensional Quantum Physics, Department of Physics, Tsinghua University, Beijing100084, China; State Key Laboratory of Low Dimensional Quantum Physics, Department of Physics, Tsinghua University, Beijing100084, China; State Key Laboratory of Low Dimensional Quantum Physics, Department of Physics, Tsinghua University, Beijing100084, China; State Key Laboratory of Low Dimensional Quantum Physics, Department of Physics, Tsinghua University, Beijing100084, China; State Key Laboratory of Low Dimensional Quantum Physics, Department of Physics, Tsinghua University, Beijing100084, China; State Key Laboratory of Low Dimensional Quantum Physics, Department of Physics, Tsinghua University, Beijing100084, China; State Key Laboratory of Low Dimensional Quantum Physics, Department of Physics, Tsinghua University, Beijing100084, China; Institute of Physics, Chinese Academy of Sciences, Beijing100190, China; School of Materials Science and Engineering, Tsinghua University, Beijing100084, China; Beijing Academy of Quantum Information Sciences, Beijing100193, China; Beijing Academy of Quantum Information Sciences, Beijing100193, China; State Key Laboratory of Low Dimensional Quantum Physics, Department of Physics, Tsinghua University, Beijing100084, China; Beijing Academy of Quantum Information Sciences, Beijing100193, China; School of Materials Science and Engineering, Tsinghua University, Beijing100084, China; State Key Laboratory of Low Dimensional Quantum Physics, Department of Physics, Tsinghua University, Beijing100084, China; Frontier Science Center for Quantum Information, Beijing100084, China; Hefei National Laboratory, Hefei230088, China; State Key Laboratory of Low Dimensional Quantum Physics, Department of Physics, Tsinghua University, Beijing100084, China; Frontier Science Center for Quantum Information, Beijing100084, China; Hefei National Laboratory, Hefei230088, China; State Key Laboratory of Low Dimensional Quantum Physics, Department of Physics, Tsinghua University, Beijing100084, China; Frontier Science Center for Quantum Information, Beijing100084, China; Beijing Academy of Quantum Information Sciences, Beijing100193, China; Southern University of Science and Technology, Shenzhen518055, China; State Key Laboratory of Low Dimensional Quantum Physics, Department of Physics, Tsinghua University, Beijing100084, China; Frontier Science Center for Quantum Information, Beijing100084, China; Beijing Academy of Quantum Information Sciences, Beijing100193, China; Hefei National Laboratory, Hefei230088, China; Beijing Academy of Quantum Information Sciences, Beijing100193, China; State Key Laboratory of Low Dimensional Quantum Physics, Department of Physics, Tsinghua University, Beijing100084, China; Frontier Science Center for Quantum Information, Beijing100084, China; Beijing Academy of Quantum Information Sciences, Beijing100193, China; Hefei National Laboratory, Hefei230088, China

**Keywords:** intrinsic magnetic topological insulator, quantized anomalous Hall resistivity, molecular beam epitaxy, quantized transport property

## Abstract

The intrinsic magnetic topological insulator MnBi_2_Te_4_ provides a feasible pathway to the high-temperature quantum anomalous Hall (QAH) effect as well as various novel topological quantum phases. Although quantized transport properties have been observed in exfoliated MnBi_2_Te_4_ thin flakes, it remains a big challenge to achieve molecular beam epitaxy (MBE)-grown MnBi_2_Te_4_ thin films even close to the quantized regime. In this work, we report the realization of quantized anomalous Hall resistivity in MBE-grown MnBi_2_Te_4_ thin films with the chemical potential tuned by both controlled *in situ* oxygen exposure and top gating. We find that elongated post-annealing obviously elevates the temperature to achieve quantization of the Hall resistivity, but also increases the residual longitudinal resistivity, indicating a picture of high-quality QAH puddles weakly coupled by tunnel barriers. These results help to clarify the puzzles in previous experimental studies on MnBi_2_Te_4_ and to find a way out of the big difficulty in obtaining MnBi_2_Te_4_ samples showing quantized transport properties.

## Teaser

The intrinsic magnetic topological insulator MnBi_2_Te_4_ provides a promising route towards high temperature quantum anomalous Hall effect. This work reports quantized transport behaviors achieved in molecular beam epitaxy-grown MnBi_2_Te_4_ thin films for the first time. The progress lays the foundation for constructing complex structures and arrays of MnBi_2_Te_4_-based quantum anomalous Hall devices for various studies and practical applications.

## INTRODUCTION

The quantum anomalous Hall (QAH) effect is a quantum Hall (QH) effect induced by spontaneous magnetization of a material and thus free from an external magnetic field [1]. After the first experimental realization in molecular beam epitaxy (MBE)-grown thin films of a magnetically doped topological insulator (TI) in 2013 [2], the QAH effect has attracted rapidly growing research interest as a route towards various other exotic quantum phases such as axion insulator and chiral topological superconductor [3,4]. Magnetically doped TIs need a rather low temperature (usually <1 K) to exhibit the QAH effect and are highly disordered due to the randomly distributed magnetic dopants [5,6]. In recent years, two kinds of intrinsic QAH systems have been discovered, namely thin films/flakes of MnBi_2_Te_4_-family compounds [7–11] and moiré superlattices of 2D materials [12–14], providing potentially cleaner and more robust alternatives to magnetically doped TIs. Particularly, the huge magnetic gap predicted in MnBi_2_Te_4_ films (∼50 meV) heralds a high-temperature QAH effect. Nearly quantized transport properties have indeed been observed in MnBi_2_Te_4_ thin flake samples up to ∼40 K [15]. However, MnBi_2_Te_4_ thin flakes (also moiré superlattices) rely on rather challenging and tricky fabrication techniques, and suffer from the irregular shape, small size and relatively low yield for quantized transport properties. The intractable thin flake samples make systematic studies and optimizations very difficult, leading to seemingly inconsistent data from different samples and measurements [11,15–20]. Moreover, to explore QAH-based novel topological quantum states [21,22] and their electronic applications, MnBi_2_Te_4_ samples should be prepared in a repeatable, controllable and scalable way so that they can construct complex and large-area heterostructures, arrays and circuits for different purposes. It is very difficult for thin flake samples to satisfy the requirement.

MBE can provide large, regular and well-controlled samples with excellent repeatability and tunability, particularly advantageous in the scalable preparation of QAH-based heterostructures. Although MBE growth of MnBi_2_Te_4_ thin films has long since been achieved, the largest Hall resistivity obtained in those samples merely reached half of the quantized value (*h*/*e*^2^) so far [10,23–27]. In this work, we achieved MBE-grown MnBi_2_Te_4_ thin films showing quantized Hall resistivity in the ferromagnetic (FM) configuration under high magnetic field. The much larger sample size and higher repeatability of sample properties enable us to systematically study their properties and clarify some puzzles in previous works on MnBi_2_Te_4_.

## RESULTS AND DISCUSSION

**Figure 1.**
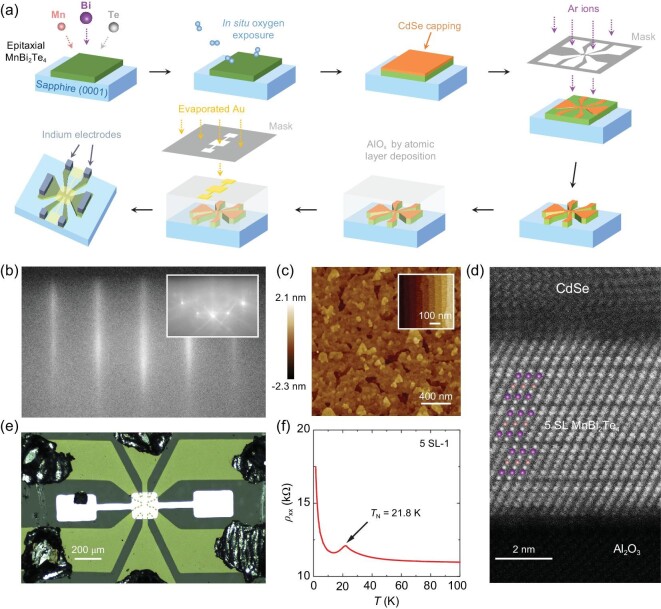
Characterizations of MnBi_2_Te_4_ thin films. (a) The schematic procedure of sample preparation. (b) RHEED patterns of an as-grown sample and its substrate (inset) in the same direction. (c) Topographies of sample 5 SL-1 with capping layer and a treated substrate (inset). (d) A typical STEM cross-sectional image of a 5-SL sample with same atom colors in (a). (e) Optical microscopy image of a typical device with the Hall bar size of 40 μm × 80 μm. The dashed line is guide to eye. (f) The *ρ_xx_*–*T* curve of sample 5 SL-1 with *V*_g_ = 0 V.

The schematic procedure of sample preparation is shown in Fig. 1a. MnBi_2_Te_4_ films are grown on sapphire (0001) substrates by co-evaporation of Mn, Bi and Te, and post-annealing at 270^o^C (see Methods, SI A and [28] for the detailed MBE growth procedure and parameters). Figure 1b shows the reflection high energy electron diffraction (RHEED) pattern of a MnBi_2_Te_4_ film (the inset is that for the sapphire substrate). The as-grown MnBi_2_Te_4_ films are electron-doped due to unavoidable occupying of Bi atoms on Mn sites [29], similar to single crystal samples. We apply *in situ* oxygen exposure and electric gating to coarsely and finely tune the Fermi level into the surface state gap, respectively [30]. The as-grown films are transferred to another chamber of the same MBE system and exposed to ∼1 × 10^−3^ mbar of O_2_ for 1 h at room temperature. The pressure resembles the O_2_ partial pressure in a glove box for exfoliating MnBi_2_Te_4_ flakes. The oxygen pressure is slightly modified for samples of different thicknesses to keep them roughly charge-neutral. Such a medium O_2_ exposure effectively reduces the carrier density of MnBi_2_Te_4_ films with little influence to their electronic structures and properties (Supplementary Figs S2 and S3). After O_2_ exposure, an 8-nm-thick cadmium selenide (CdSe) capping layer is deposited onto the films to avoid uncontrolled charge doping in the following fabrications and measurements. The atomic force microscopy and high-resolution scanning transmission electron microscopy (STEM) images of a nominally five-septuple-layer (5-SL) MnBi_2_Te_4_ sample with CdSe capping layer are shown in Fig. 1c and d, respectively. The film is mainly composed of one terrace with uniform thickness, distributed with small islands and depressions mainly within 1-SL height.

The MnBi_2_Te_4_ films are lithographed by using argon ion milling into Hall bars (40 μm × 80 μm) through a molybdenum mask. An AlO_x_ dielectric layer is then grown on the samples by using atomic layer deposition and the top-gate electrode is deposited on it through another molybdenum mask (the upper limit of the sample size is determined by the quality of the dielectric layer). The false-color photo of a final device is displayed in Fig. 1e. The sample temperature is kept at <60^o^C during the whole top-gate structure fabrication procedure to avoid sample degradation. The low temperature tolerance may come from the oxygen introduced in the films. Different devices on a 3 mm × 5 mm sample show similar transport properties (Supplementary Fig. S9), indicating the excellent homogeneity and repeatability of the MBE-grown MnBi_2_Te_4_ samples. Figure 1f displays the longitudinal resistivity (*ρ_xx_*)–temperature (*T*) curve of a 5-SL MnBi_2_Te_4_ thin film (with the gate voltage *V*_g_ = 0 V), which exhibits a clear cusp at 21.8 K corresponding to the Néel temperature (*T*_N_) and an insulating behavior at lower temperatures [31,32].

**Figure 2.**
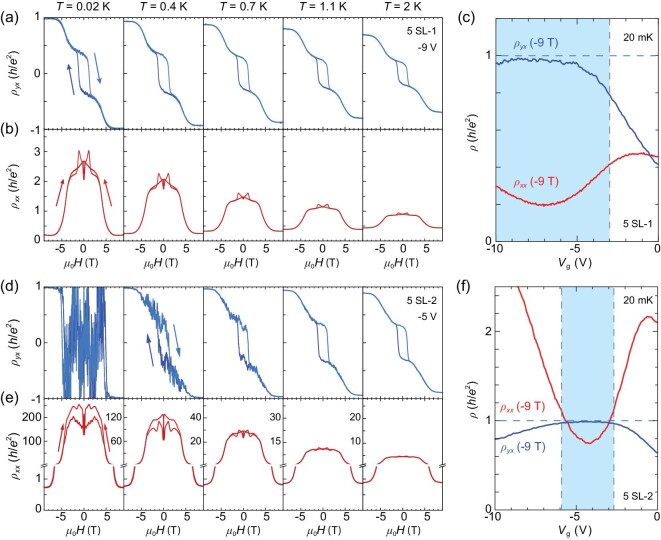
Magneto-transport measurements of 5-SL samples with different post-annealing time. (a and b) The *ρ_yx_*–*μ*_0_*H* and *ρ_xx_*–*μ*_0_*H* curves of sample 5 SL-1 (post-annealed for 30 min) at selected temperatures of ≤2 K, respectively. (c) The anti-symmetrized *ρ_yx_*–*V*_g_ and symmetrized *ρ_xx_*–*V*_g_ curves of sample 5 SL-1 under −9 T at 20 mK. (d–f) The transport results of sample 5 SL-2 (post-annealed for 120 min) with the same measurements as sample 5 SL-1 [corresponding to (a–c) respectively].

Figure 2a and b shows the magnetic field (*μ*_0_*H*) dependences of the Hall (*ρ_yx_*) and longitudinal (*ρ_xx_*) resistivities, respectively, of a 5-SL sample (at charge-neutral point) measured at five different temperatures. Figure 2c displays the *ρ_yx_*–*V*_g_ and *ρ_xx_*–*V*_g_ curves at *T* = 0.02 K and *μ*_0_*H* = −9 T. The sample was post-annealed at 270^o^C for 30 min after MBE growth (referred to below as 5 SL-1). The *ρ_yx_*−*μ*_0_*H* and *ρ_xx_*−*μ*_0_*H* curves have the typical shapes of MnBi_2_Te_4_: the high-field (>6.5 T) and low-field (<3 T) parts of the curves roughly correspond to MnBi_2_Te_4_ in the FM and antiferromagnetic (AFM) configurations, respectively. In the FM state around ±9 T, |*ρ_yx_|* and *ρ_xx_* always evolve inversely, with magnetic field, gate voltage and temperature, which is a signature of the dissipationless QH or QAH edge state. The former one is excluded by the absent transport evidence of Landau levels, such as Shubnikov de Haas peaks, and also the low carrier mobility (at most several hundred cm^2^/V⋅s, as shown in Supplementary Fig. S11c). Therefore, the QAH state dominates the high-field transport properties. At 0.02 K, |*ρ_yx_|* reaches 0.98 *h*/*e*^2^ and *ρ_xx_* drops to 0.20 *h*/*e*^2^ at ±9 T, exhibiting a decent quantization. Descendible deviation of *ρ_yx_* from quantization is observed at 0.4 K (0.93 *h*/*e*^2^). It is difficult to accurately estimate the activation gap from the *σ_xx_*–*T*^−1^ curve (Supplementary Fig. S14). But according to Fig. 2a and b, the gap size should be at the same level as that of Cr-doped (Bi, Sb)_2_Te_3_ films and much lower than that of good MnBi_2_Te_4_ flake samples. In the AFM state at low field (between ±3 T), the *ρ_yx_*–*μ*_0_*H* curves exhibit a hysteresis loop, with the coercive field (*H*_c_) of ∼1.1 T and zero field *ρ_yx_* of ∼0.34 *h*/*e*^2^ at 0.02 K (Fig. 2a). *ρ_xx_* at high field decreases with decreasing temperature, as expected in a QAH system; meanwhile, at low field, *ρ_xx_* increases with decreasing temperature, showing an insulating behavior (Fig. 2b).

We systematically investigate the thickness-dependent transport properties of the MnBi_2_Te_4_ films from 2 to 7 SL, all post-annealed at 270^o^C for 30 min, as shown in Supplementary Fig. S5. The 4-SL to 6-SL samples all show a nearly quantized *ρ_yx_* and a *ρ_xx_* minimum at high field in both the *μ*_0_*H*- and *V*_g_-dependent curves, indicating the QAH states in the FM state. No signatures of the QAH state are observed in the 2-SL and 3-SL films. The absence of the QAH state in the 3-SL film, which ought to be in the QAH regime according to early works [7,11], may be attributed to the influence of oxidation [33]. In the 7-SL film, the signatures of the QAH state can hardly been distinguished due to parallel conduction channels.

We found that elongated post-annealing significantly changes the transport properties of the films. Figure 2d and e displays the *ρ_yx_*−*μ*_0_*H* and *ρ_xx_*−*μ*_0_*H* curves, respectively, of a 5-SL MnBi_2_Te_4_ film with post-annealing at 270^o^C for 120 min (referred to as 5 SL-2) measured at different temperatures. The annealing was carried out in Te flux to avoid Te desorption. Notably, |*ρ_yx_*| of the sample reaches full quantization (0.99 *h*/*e*^2^) at 0.02 K at high field (the strong noise in the *ρ_yx_*−*μ*_0_*H* curve between ±5 T is due to the huge *ρ_xx_*, which will be discussed below). The Hall resistivity remains at 0.98 *h*/*e*^2^ at 0.4 K, 0.97 *h*/*e*^2^ at 0.7 K and still 0.89 *h*/*e*^2^ even at 2 K. Obviously, the *ρ_yx_* quantization at high field becomes more robust to temperature, comparable to some MnBi_2_Te_4_ thin flake samples. On the other hand, *ρ_xx_* is significantly enhanced, rather than reduced as expected in a good QAH sample, by the elongated annealing. The high-field *ρ_xx_* of the sample is 0.73 *h*/*e*^2^ at ±9 T, 0.02 K, and rises with increasing temperature, suggesting that the ground state is still the QAH phase. The low-field *ρ_xx_* at 0.02 K becomes extremely large (the measured *ρ_xx_* of ∼200 *h*/*e*^2^ is not accurate due to the too large resistance) and drops rapidly with increasing temperature (∼5 *h*/*e*^2^ even at 2 K), exhibiting a typical insulating behavior. It suggests the absence of dissipationless chiral edge states in the sample at low field, which will be discussed later.

**Figure 3.**
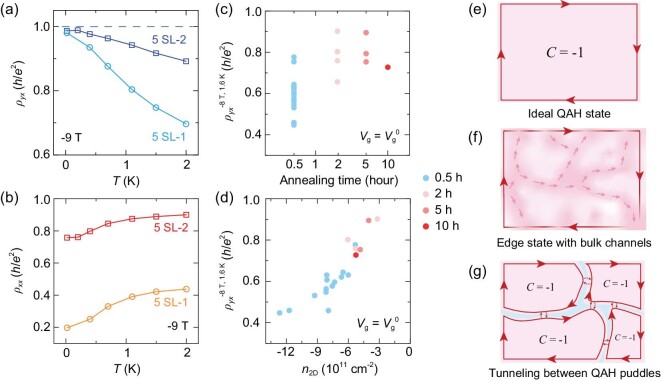
Transport properties of films with different post-annealing time in the ferromagnetic configuration. (a and b) The comparisons of *ρ_yx_*–*T* and *ρ_xx_*–*T* curves of samples 5 SL-1 and 5 SL-2 under −9 T at the charge-neutral point, respectively. (c and d) The relations between *ρ_yx_* (−8 T) at 1.6 K and post-annealing time, *ρ_yx_* (−8 T) at 1.6 K and carrier density (*n*_2D_), respectively. All the data are taken at the charge-neutral point (*V*_g_ = *V*_g_^0^) of each sample. (e–g) The schematic of three different scenarios of QAH state in realistic samples. Pink, purple and cyan regions represent QAH insulators, conductive bulk channels (with purple arrows) and insulating regions, respectively. Red solid and dashed lines represent edge states and tunneling processes between edge states, respectively.

The effect of annealing duration can be clearly seen in the *ρ_yx_*−*V*_g_ and *ρ_xx_*−*V*_g_ curves measured at −9 T and 0.02 K of the 5 SL-1 (Fig. 2c) and 5 SL-2 (Fig. 2f) samples, and also in their temperature dependences of *ρ_yx_* (Fig. 3a) and *ρ_xx_* (Fig. 3b) at −9 T. Obviously, elongated annealing drives *ρ_yx_* closer to the quantized value (*h*/*e*^2^) but makes *ρ_xx_* further away from zero at high field. Longer post-annealing duration time (at 270^o^C) does not obviously change the sample properties further, as shown in Fig. 3c. So 120 min of post-annealing is selected representatively. A similar annealing effect is also observed in the films of other thicknesses (Supplementary Fig. S14).

In a well-quantized QAH film, the dissipationless chiral edge channel surrounding the whole sample contributes to quantized *ρ_yx_* and vanishing *ρ_xx_* (see Fig. 3e). With dissipative conduction channels of residual bulk carriers, *ρ_xx_* becomes non-zero and *ρ_yx_* drops to below the quantized value (Fig. 3f). The early results on the QAH effect and the data of the MnBi_2_Te_4_ film with 30 min of annealing (Fig. 2a and b) are all consistent with this case. It thus looks confusing that the sample with 120 min of annealing has a perfectly quantized *ρ_yx_* below 0.4 K but, at the same time, keeps a large *ρ_xx_* at high field (Fig. 2d and e). The quantized *ρ_yx_* accompanied by a significant *ρ_xx_* was ever observed and discussed in 2D electron systems, known as the ‘quantized Hall insulator’, which is composed of weakly coupled QH regions (puddles) [34], each surrounded by dissipationless QH edge states. The tunneling between the neighboring QH puddles contributes to a large *ρ_xx_* of the whole sample, which is determined by the transmission probability (Fig. 3g) [35]; meanwhile, *ρ_yx_* can still remain the quantized value. Although the MnBi_2_Te_4_ films with elongated annealing have a QAH ground state at high field, not really an insulator, the high residual *ρ_xx_* can be understood with this picture. Namely, the samples are composed of well-quantized QAH puddles with the tunneling between the puddles giving a large *ρ_xx_* of the whole sample.

Through ordinary Hall effect measurements above *T*_N_, we found that the MnBi_2_Te_4_ films with longer-time post-annealing have a lower carrier density at the charge-neutral point (Fig. 3d). It is still unclear what happens in the films with longer-time annealing. One possibility is that long-time annealing aggregates the defects or impurities that induce the chemical potential fluctuation in a MnBi_2_Te_4_ film [36], e.g. Mn atoms occupying Bi sites. As a result, most areas of the film become cleaner with fewer chemical potential fluctuations, acting as high-quality QAH puddles. But at their boundaries where defects aggregate, the QAH phases are destroyed and the dissipationless edge states of QAH puddles can only tunnel through there, contributing to the non-zero *ρ_xx_*. Therefore, the residual *ρ_xx_* is expected to be reduced in MnBi_2_Te_4_ films of smaller size, which includes fewer QAH puddles. Our preliminary result indeed shows that a MnBi_2_Te_4_ device of smaller size exhibits a lower *ρ_xx_* under high field than a device of larger size (Supplementary Fig. S12), while a systematic size-dependent transport study is needed to confirm it.

**Figure 4.**
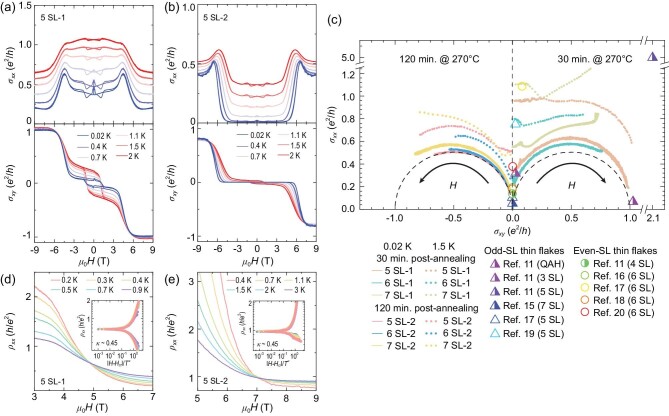
Quantum phase transitions in MnBi_2_Te_4_ thin films. (a and b) The temperature evolutions of *σ_xx_*–*μ*_0_*H* and *σ_xy_*–*μ*_0_*H* curves of sample 5 SL-1 and sample 5 SL-2, respectively. (c) Phase diagram depicted by the conductivity tensor. MBE-grown thin films are labeled with different colors. The solid and dotted lines represent the data taken at the base temperature and 1.5 K, respectively. The left and right parts correspond to the data from the long- and short-time post-annealed samples, respectively. Both sides are *σ* evolutions with increasing magnetic field (indicated by the black arrow). The triangles and circles represent conductivity tensor data of odd- and even-SL exfoliated thin flakes taken from previous reports around 1.5 K at 0 T, respectively. The aspect ratio of length (*L*) to width (*W*) of each device is used to calculate conductivities when they are shown (hollow symbols). If not, *L/W* = 1 is used (symbols colored half). The black dashed semicircle is guide to eye. (d and e) The quantum phase transitions near *H*_cr_s of sample 5 SL-1 and sample 5 SL-2, respectively. The insets show the scaling analyses.

Figure 4a and b displays the *μ*_0_*H* dependences of the longitudinal (*σ_xx_*) and Hall (*σ_xy_*) conductivities measured at different temperatures of the 5-SL films with 30 and 120 min of post-annealing, respectively. At high field, the former sample has nearly quantized *σ_xy_*; meanwhile, the latter one has *σ_xy_* obviously lower than *e*^2^/*h* due to the larger *ρ_xx_*, despite the fully quantized *ρ_yx_*. The ground states of the films can be checked by the tendency of the conductivity tensor (*σ_xy_, σ_xx_*) evolution with increasing magnetic field and decreasing temperature (the flow charts), as shown in Fig. 4c. Here, the data of 5-SL, 6-SL and 7-SL films are plotted and the left and right parts correspond to the data from the long- and short-time post-annealed samples, respectively. Similar to early results of the QH and QAH effects [37–40], the conductivity tensors tend to flow towards (0, 0) or (*e*^2^*/h*, 0) points at low temperature. The (0, 0) point is the insulating phase (also known as the Hall insulator) and the (*e*^2^*/h*, 0) point is the QAH or QH phases [41–44].

In the FM configuration (high field), the conductivity tensors of the films all flow towards the (*e*^2^*/h*, 0) points with increasing magnetic field (indicated by the black arrow) and decreasing temperature, which indicates a QAH ground state (the QH phase is excluded as discussed above). In the AFM configuration (low field), the conductivity tensors all flow to the (0, 0) point with decreasing magnetic field (the opposite direction of the black arrow) and temperature, regardless of odd- or even-SL films, which indicates an insulating ground state as directly observed in Fig. 2b and e. The *ρ_xx_*−*μ*_0_*H* curves of the two 5-SL samples measured at different temperatures all cross at one point, signifying a quantum phase transition from the QAH state (high field) to the insulating state (low field). A scaling analysis near the critical magnetic field (*H*_cr_) is shown in the insets of Fig. 4d and e, where the relation between *ρ_xx_* and the scaling variable (*H* – *H*_cr_)/*T^κ^* are plotted. *ρ_xx_* collapses into two branches contributed by two sides of *H*_cr_, respectively, with the fitted scaling exponent *κ* ∼ 0.45, identical to that for the phase transitions in QH systems [45].

The insulating behavior in the odd-SL films at low field looks inconsistent with the prediction that MnBi_2_Te_4_ in the AFM configuration is a Chern insulator in the odd-SL films [7–9]. One possible cause is the thickness fluctuations of films. The 1-SL islands and depressions as shown in Fig. 1c make an odd-SL QAH film mixed with even-SL axion insulator regions, which could elevate *ρ_xx_*. However, according to early theoretical and experimental results on magnetically modulation-doped TI films [46], even when half of a QAH film becomes an axion insulator, *ρ_xx_* only increases to ∼1 *h*/*e*^2^, which is much lower than the observed *ρ_xx_* of up to hundreds of *h*/*e*^2^ in the MnBi_2_Te_4_ films with 120 min of annealing. Actually, we found that the measured *ρ_xx_* of odd-SL films is not sensitive to the coverage of the even-SL regions (Supplementary Fig. S13). The zero-magnetic-field insulating state has also been reported in many odd-SL MnBi_2_Te_4_ flakes that show quantized *ρ_yx_* at high field, without thickness fluctuation [15,17,19,47]. The conductivity tensor data of MnBi_2_Te_4_ thin flakes at zero field around 1.5 K are also plotted in Fig. 4c for comparison. Most of the data are near the (0, 0) point, suggesting an insulating ground state. The insulating ground state is confirmed by the huge resistivity observed in our MBE films. Only in very rare odd-SL flake samples has the QAH effect at zero magnetic field been observed [11].

Another possibility is that many AFM domains exist in the films around zero magnetic field, instead of the single AFM domain case assumed by the theoretical work predicting the QAH effect. Antiferromagnetic domains have been observed in MnBi_2_Te_4_ single crystals by using magnetic force microscopy with the typical size of ∼10 μm [48,49]. Since the domain size is much larger than the width of the QAH edge states in MnBi_2_Te_4_ (∼1 μm) [20], the sample can be considered as a network of chiral edge states, which usually gives a *ρ_xx_* of the same order of magnitude as *h*/*e*^2^ [50,51]. Again, the huge *ρ_xx_* observed in the samples with 120 min of annealing suggests absence of the chiral edge state network. However, the magnetic structure of the MnBi_2_Te_4_ thin films/flakes might be different from single crystals due to the low thickness. There are other possibilities, e.g. the influences of antisite defects [52], that might also lead to the insulating behavior in MnBi_2_Te_4_ thin films/flakes at zero field. The present data are not enough to decide which mechanism dominates. Nevertheless, the huge *ρ_xx_* at zero magnetic field and its sensitivity to the post-annealing condition may help in understanding the large fluctuations in the sample properties of MnBi_2_Te_4_ thin flakes. Besides, considering that the QAH state is probably absent in most MnBi_2_Te_4_ samples at zero magnetic field, whatever the reason is, it is reasonable that previous angle-resolved photoemission spectroscopy measurements on MnBi_2_Te_4_ (on large samples at zero magnetic field) failed to clearly demonstrate the magnetic gap opening as predicted theoretically [22].

## CONCLUSION

In summary, we have achieved MBE-grown MnBi_2_Te_4_ thin films exhibiting quantized Hall resistivity at high magnetic field. Both even- and odd-SL films show insulating behavior around zero magnetic field. Elongating the post-annealing time is found to significantly enhance the temperature to reach *ρ_yx_* quantization (at high field) but at the same time increase the residual *ρ_xx_*, indicating formation of QAH puddles with their chiral edge states tunneled with each other. The information obtained from the MBE-grown films with good repeatability may help to clarify the complexity in the experimental results on MnBi_2_Te_4_ and improve the material for explorations of the high-temperature QAH effect and other novel topological quantum effects.

## METHODS

### Thin film growth

MnBi_2_Te_4_ thin films are grown on treated sapphire (0001) substrates in an ultrahigh-vacuum MBE system with base pressure ≤2.0 × 10^−10^ mbar. The commercial substrates are annealed in a tube furnace at 1100^o^C for 3 h under an O_2_ atmosphere to obtain flat terraces on the surfaces. The treated substrates are degassed at 400^o^C for 30 min before growth. High-purity Mn (99.9998%), Bi (99.9999%) and Te (99.9999%) are co-evaporated with commercial Knudsen cells. The post-annealing process at growth temperature for 0.5−10 h is implemented to further improve the sample quality. (If the annealing time is ≥2 h, Te flux is applied to avoid Te desorption.) After growth, the samples are *in situ* exposed to oxygen with different pressures for different thicknesses and then an 8-nm CdSe layer is capped on the top at room temperature. Before the following fabrication, the topography is scanned by using atomic force microscopy (Bruker, Innova). See more details in SI A.

### Fabrication

The MnBi_2_Te_4_ films are lithographed by using argon ion milling into Hall bars (40 μm × 80 μm) through a molybdenum mask. Then, a 40-nm AlO_x_ layer is grown on the samples as a gate dielectric by using atomic layer deposition. The top-gate electrode of 5 nm Ti/20 nm Au is deposited on it through another molybdenum mask. Indium is applied as Hall bar electrodes.

### Transport measurements

Magneto-transport measurements are performed in a commercial dilution fridge Oxford Instrument Triton 400 with a base temperature of <20 mK. Due to the large resistance at low temperature, we use delta mode, combining source meter and voltmeter. The DC current is applied by using the Keithley 6221 digital source meter. The voltage drop is measured by using the Keithley 2182A voltmeter. At low temperature, the delay time between the start of the DC current being applied and the voltage reading is from 1.6 to 5.1 s for different samples. The longitudinal and Hall voltage drops *V_xx_* and *V_yx_* are detected separately in different magnetic field sweeps. For samples 5 SL-1, 6 SL-1 and 7 SL-1, the source-drain currents used in longitudinal (*I_xx_*) and Hall (*I_yx_*) measurements are both 1 nA. For samples 4 SL, 5 SL-2, 6 SL-2 and 7 SL-2, *I_xx_* and *I_yx_* are 1 and 10 nA, respectively. The current remains unchanged during the variation in temperature and gating. The top-gate (AlO_x_ dielectric) voltages are applied by using a Keithley 2400 multimeter.

## Supplementary Material

nwad189_Supplemental_File
