# Supplementary material for: Quantized anomalous Hall resistivity achieved in molecular beam epitaxy-grown MnBi2Te4 thin films
Source: Natl Sci Rev. 2023 Jul 4;11(2):nwad189. doi: 10.1093/nsr/nwad189 (PMC10776363; doi:10.1093/nsr/nwad189)
Supplement: nwad189_Supplemental_File [file nwad189_supplemental_file.pdf]

Supplementary Information

Quantized anomalous Hall resistivity achieved in molecular beam epitaxy-grown $MnBi_2Te_4$ thin films

Yunhe Bai[1]*, Yuanzhao Li[1]*, Jianli Luan[1]*, Ruixuan Liu[1]*, Wenyu Song[1], Yang Chen[1], Peng-Fei Ji[1], Qinghua Zhang[7], Fanqi Meng[6], Bingbing Tong[3], Lin Li[3], Yuying Jiang[1], Zongwei Gao[3], Lin Gu[6], Jinsong Zhang[1,2,5], Yayu Wang[1,2,5], Qi-Kun Xue[1,2,3,4], Ke He[1,2,3,5†], Yang Feng[3†], and Xiao Feng[1,2,3,5†]

[1]*State Key Laboratory of Low Dimensional Quantum Physics, Department of Physics, Tsinghua University, Beijing 100084, China*

[2]*Frontier Science Center for Quantum Information, Beijing 100084, China*

[3]*Beijing Academy of Quantum Information Sciences, Beijing 100193, China*

[4]*Southern University of Science and Technology, Shenzhen 518055, China*

[5]*Hefei National Laboratory, Hefei 230088, China*

[6]*School of Materials Science and Engineering, Tsinghua University, Beijing 100084, China*

[7]*Institute of Physics, Chinese Academy of Sciences, Beijing 100190, China*

* *These authors contributed equally to this work.*

† *Corresponding author. Email:* kehe@tsinghua.edu.cn (K. H.); fengyang@baqis.ac.cn (Y. F.); xiaofeng@mail.tsinghua.edu.cn (X. F.)

Contents:

SI A: Details in MBE growth 1

SI B: The role of in-situ oxygen exposure 1

SI C: Magneto-transport properties of different-thick films 8

SI D: High repeatability and scalability of MBE-grown films 10

SI E: More STEM cross-sectional images of MBE-grown films 12

SI F: Statistical results on different post-annealing time and coverages 12

SI G: Quantum phase transitions in films with different thicknesses 17

SI A: Details in MBE growth

$MnBi_2Te_4$ thin films are grown on 0.5-mm sapphire (0001) substrates in a commercial MBE system (Omicron Lab-10) with a base pressure better than 2×10^{-10} mbar. High purity Mn (99.9998%), Bi (99.9999%), Te (99.9999%) are co-evaporated from commercial Knudsen cells with temperatures of 540°C, 522°C and 251°C, respectively. The flux ratio of Te:(Mn + Bi) is greater than 10 to prevent Te deficiency. A two-step growth sequence is adopted, i.e. the substrate temperature is set to 240°C for 2.5 minutes firstly, then raised to 270°C. Post-annealing is implemented at growth temperature for 0.5~10 hours to further improve sample quality (If the annealing time is longer than 2 hours, Te flux is applied to avoid Te desorption). The temperature is calibrated by a pyrometer (LumaSense Technologies, IGA 6/23) through a vacuum viewport, located right below the sample. After growth, the sample is transferred to another chamber of the same MBE system, connected with a high-purity oxygen cylinder (99.9995%), for in-situ oxygen exposure at room temperature lasting for an hour. The oxygen pressure is tuned by a variable leak valve and monitored by a wide-range vacuum gauge (ATMION®). Then, an 8-nm CdSe layer is capped on the top at room temperature. The topography is scanned by an atomic force microscope (Bruker, Innova), and no obvious difference in topography has been observed between samples with and without capping layer. The growth rate of MBE-grown $MnBi_2Te_4$ films is about 0.1 SL/minute, calibrated by the growth time and thickness of sample.

SI B: The role of in-situ oxygen exposure

To realize the quantized Hall resistivity, a finely tuned Fermi level residing in the magnetic gap is necessary. In this work, we employ in-situ oxygen exposure and electric gating to coarsely and finely tune the Fermi level, respectively. Before going any further, we first examine the transport behaviors in as-grown samples. In a typical as-grown 5-SL sample on sapphire (0001) substrate with an 8-nm CdSe capping layer, the Fermi level is far from the charge-neutral point (CNP) manifested by the metallic behavior of

temperature-dependent R_{xx} above Néel temperature (T_N) and relatively small R_{xx} (Fig. S1a). The insulating behavior below T_N can be understood by the localization of massive Dirac fermions, since the AFM order has been constructed. Moreover, the slopes of R_{yx}-μ_0H curves also indicate a high n-type carrier density (Fig. S1b).

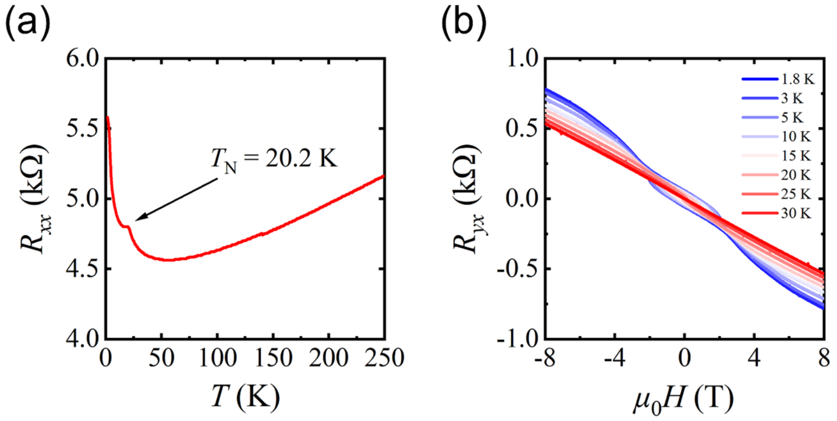

Figure S1. Transport measurements of an as-grown 5-SL sample on sapphire (0001) with an 8-nm capping layer. (a) The T dependence of R_{xx}. (b) The temperature evolution of R_{yx}–μ_0H curve.

Due to the high n-type carrier concentration, a top gate alone is insufficient for effective carrier tuning even in 3-SL as-grown $MnBi_2Te_4$ films. Things may get worse with thicker films since the electric field is further screened. Thus call for extra tuning capability with little influence on sample quality. Oxygen doping, which has been employed to tune the Fermi level in Bi_2Se_3 system [1], and antimony (Sb) doping, a convenient band structure-engineering method in $(Bi,Sb)_2Te_3$ system, have both been considered. In Sb-doped $MnBi_2Te_4$, the difference of in-plane lattice constants within each SL gets smaller, lowering the formation energy of cation-antisite defects with Mn [2]. The Sb doping may not only aggravate the spatial inhomogeneity and reduce the mobility gap, but also lead to more magnetic disorder and a lower saturation moment [3,4]. Considering the potential disadvantages, we tried in-situ oxygen exposure after MBE growth.

In-situ RHEED and ARPES results

To directly show the impact of O_2 exposure, we investigated the variations in the surface structure and electronic band structure of a 4-SL film, grown on a niobium-doped $SrTiO_3$ (111), with gradually increasing O_2-exposure time by in-situ RHEED and ARPES at room temperature. With a low O_2 pressure, about 10^{-4} mbar for an hour, no obvious degradation was observed in RHEED patterns (Fig. S2a). Meanwhile, a gradual shift of Fermi level with increasing exposure time (analogous to different O_2 pressures with identical exposure time) could be clearly seen in Figs. S2b and S2c, without other apparent changes in the band structure. The mechanism leading to the p-type doping is unclear. One possible explanation is that O_2 molecules occupy the Te vacancy sites and capture extra electrons, resulting in the neutral-charged band, as predicted by the theoretical calculation [5]. Some of recent works suggested that oxygen may deteriorate the quality of $MnBi_2Te_4$ samples even leading to decomposition [6–9]. While, in our films with in-situ O_2 exposure (e.g. sample 5 SL-1), a well quantized ρ_{yx} with a small

residual ρ_{xx} can still be achieved in the FM configuration (Fig. 2c). The difference between previous works and our report might be the rather low O_2 pressure we used (resembles the O_2 partial pressure in a glove box for exfoliating $MnBi_2Te_4$ flakes), which need further investigation.

We stress that the results of the sample grown on niobium-doped $SrTiO_3$ (111) for ARPES measurements (Figs. S2b and S2c), can only be understood qualitatively. It should be noted that the bulk carrier density could vary quantitatively between films on different substrates due to the distinct work functions. However, the shift of Fermi level with O_2 exposure can still be demonstrated clearly in Fig. S2. In order to realize quantized transport behaviors on insulating substrates, e.g. sapphire, the O_2 pressure should be optimized in series of transport measurements.

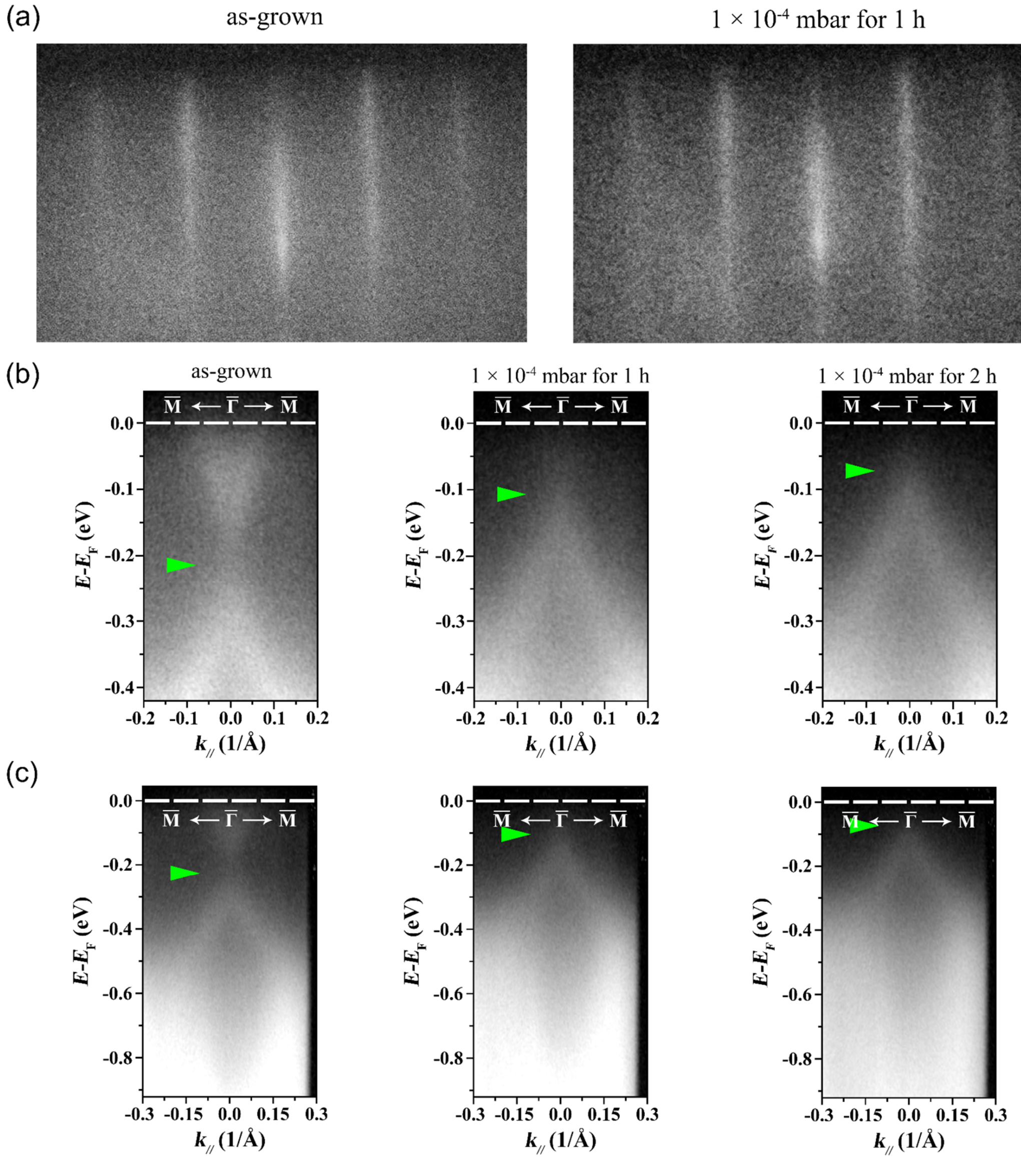

Figure S2. In-situ characterizations before and after O_2 exposure. (a) RHEED patterns of an as-grown 4-SL $MnBi_2Te_4$ film on niobium-doped $SrTiO_3$ (111) before (left) and after (right) O_2 exposure. (b, c) The evolutions of band structure with gradually increasing O_2-exposure time (from left to right) in the range of 0.5 eV, 1 eV near Fermi level, respectively. The green triangles point to the top of valence bands for visual comparison.

Results of the systematically controlled transport experiment

To more quantitatively elucidate the carrier tuning by in-situ oxygen exposure, a systematically controlled transport experiment of several 4-SL $MnBi_2Te_4$ thin films on insulating $SrTiO_3$ (111) substrates with different exposed O_2 pressures was performed. Usually, an extra carrier doping could be induced during the fabrication process, e.g. atomic layer deposition (ALD) of dielectric layer. To get rid of the impact from the fabrication process, as-grown films were capped with 10-15 nm CdSe and scratched by hand directly into a Hall bar shape for measurements. Due to the absence of top gate, insulating $SrTiO_3$ (111) was chosen as the substrate instead of sapphire (0001) used in the main text, in order to give play to the tunable back gate for more information.

The transport results are shown in Fig. S3, with exposed O_2 pressure labeled at the top of each column. It can be clearly seen that ρ_{xx} (0 T) and the absolute value of slopes of ρ_{yx}–μ_0H curves get larger with higher oxygen pressure (Figs. S3a and S3b). With a low O_2 pressure (the first and second columns in Fig. S3b), the slopes of ρ_{yx}–μ_0H curves are insensitive to back-gate tuning, indicating a high carrier density. For a quantitative comparison, the carrier density and mobility of each sample near $V_g = 0$ are extracted from the ordinary Hall effect at 25 K or 30 K (above T_N), at which the magnetization has little influence. As shown at the bottom, the n-type carrier density is reduced with oxygen exposure. Meanwhile, the mobility does not show a significant degradation, at least when the pressure is no more than 10^{-3} mbar lasting for an hour. With 1 atm. oxygen pressure lasting for an hour (as shown in the last column), the carriers can be tuned from n-type to p-type both under zero field and high field (> 6 T) at 2 K. However, it is more likely a two-channel contribution rather than an uniform change in bulk, that is to say, p-type carriers come from top surface and n-type carriers come from bottom surface which is mainly tuned by the back gate [10].

Considering about the extra p-type doping from our fabrication process, experimentally, the optimized exposed pressure of oxygen applied to 5-SL, 6-SL and 7-SL films on sapphire (0001) in the main text is around 1×10^{-3} mbar lasting for an hour. For thinner films, e.g. 3-SL or 4-SL films, a lower pressure is applied.

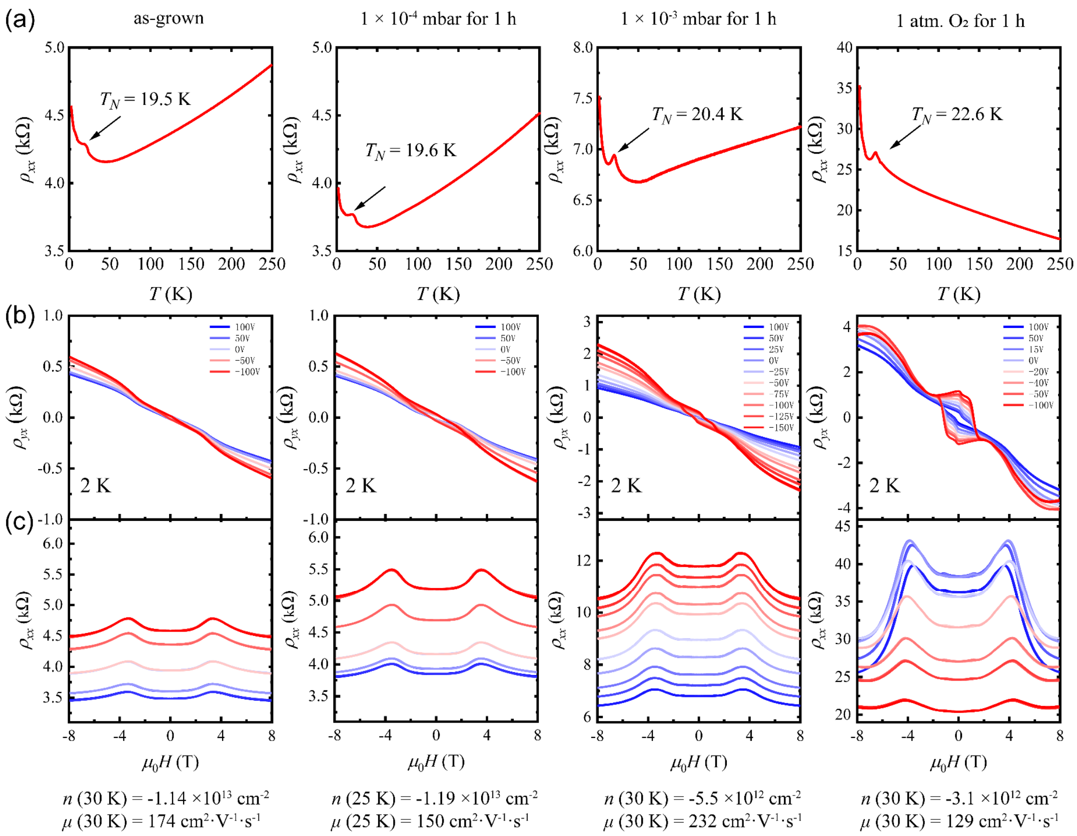

Figure S3. Transport measurements of 4-SL $MnBi_2Te_4$ thin films on high-resistance $SrTiO_3$ (111) substrates with different exposed O_2 pressures. Each column represents a sample, the exposed O_2 pressure of which is labeled at the top. (a) The ρ_{xx} (0 T)–T curves of different samples with $V_g = 0$ V. (b, c) The ρ_{yx}–$\mu_0 H$, ρ_{xx}–$\mu_0 H$ curves of different samples under various V_gs measured at 2 K. The carrier density and mobility extracted above T_N near $V_g = 0$ are shown at bottom, respectively.

ToF-SIMS measurements on $MnBi_2Te_4$ thin films

The time-of-flight secondary ion mass spectrometry (ToF-SIMS) measurements on capped 5-SL $MnBi_2Te_4$ films with and without in-situ oxygen exposure (1×10^{-3} mbar for an hour as in the main text) were applied for further chemical analysis. ToF-SIMS analysis was performed by Bi^+ primary ion beam at 30 keV over a 100 × 100 μm^2 analyzed area and the sputtering was achieved using Cs^+ ion beam at 500 eV over 400 × 400 μm^2. Negatively charged secondary ions were collected in the depth profiling. For data analysis, normalization to total ion intensity is done to remove variance in the data that may not related to actual chemical differences, e.g. instrumental conditions, sample charging and topography. The interface of capping layer and film is roughly defined as the half-maximum position of Te^- concentration. As shown in Fig. S4a, there is no obvious difference in Bi^-, Te^- and $CdSe^-$ profiles between two samples. However, the O^- concentration in the CdSe and $MnBi_2Te_4$ layers is indeed enhanced in the sample with O_2 exposure. Combined with ARPES and transport results, the reduction of electron carriers can be undoubtedly attributed to in-situ O_2 exposure.

The ToF-SIMS normalized profiles of the 5-SL $MnBi_2Te_4$ film with O_2 exposure at different areas are shown in Fig. S4b. No obvious difference in O^-, Te^-, Bi^- and $CdSe^-$ profiles among the three different areas has been observed, indicating an in-plane homogeneity. A linear scale plot of O^-/total ion intensity normalized profile of the sample is shown in Fig. S4c (a zoomed-in view of O^- normalized profile in Fig. S4b), which represents the variation of O^- concentration at different depth. A higher O^- normalized intensity near 0 s and that in sapphire (0001) could be attributed to surface oxidation by air and O element in the substrate, respectively. In-situ oxygen exposure is taken at the interface of CdSe capping layer and film, where a local maximum of O^- concentration exists. With increasing distance (sputtering time) from the CdSe/$MnBi_2Te_4$ interface, O^- normalized intensity gradually decays, indicating that the $MnBi_2Te_4$ surface is more affected by oxygen exposure than the inside of the film. Recent works on $MnBi_2Te_4$ films exposed to air claim that the oxidation occurs at only the top 2 SLs [8,9], comparable with the ToF-SIMS result. Probably, oxidation mainly occurs at the surface in our $MnBi_2Te_4$ films. On the other hand, according to the ToF-SIMS results, the Fermi energy of a $MnBi_2Te_4$ film near the sapphire substrate may also be shifted by the oxygen from the substrate. It might explain why we are able to observe quantized transport behaviors with only top gating, i.e. the Fermi energy of almost the whole film (only 4-SL- to 7-SL-thick in this work) is shifted by oxygen from the top and bottom surfaces together.

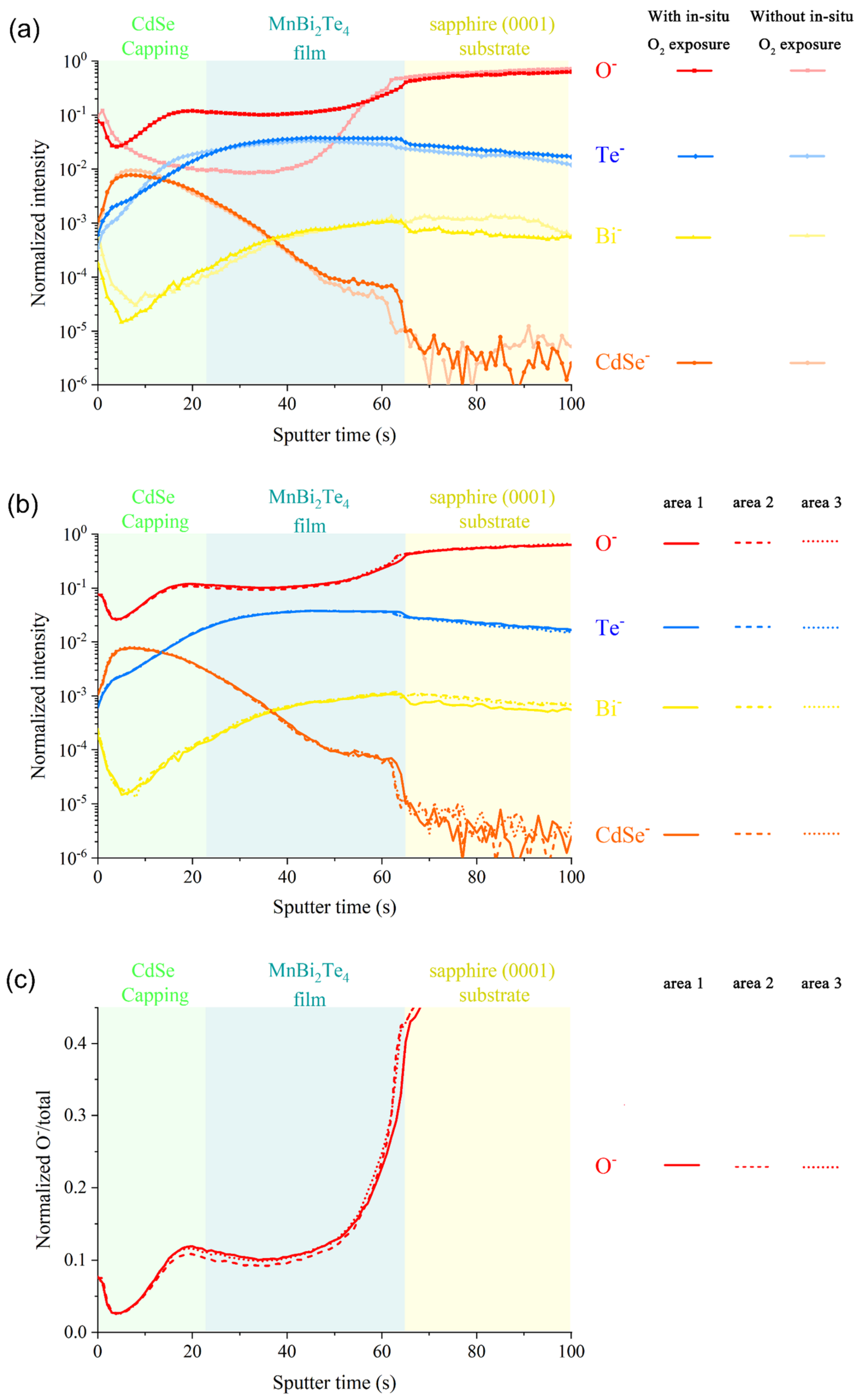

Figure S4. ToF-SIMS results of $MnBi_2Te_4$ thin films grown on sapphire (0001). (a) ToF-SIMS normalized profiles of 5-SL $MnBi_2Te_4$ films with and without in-situ O_2 exposure. (b) ToF-SIMS normalized profiles of the 5-SL $MnBi_2Te_4$ film with in-situ oxygen exposure at different areas. (c) A zoomed-in view of O^- normalized profiles in (b).

SI C: Magneto-transport properties of different-thick films

The thickness of MBE-grown thin films depends on growth time and elements flux, which provides a convenient way to systematically investigate properties with different thicknesses. MBE-grown $MnBi_2Te_4$ films are intractable to control the precise thickness as thin flakes exfoliated from single crystals, i.e. the random islands and depressions are nearly inevitable, as shown in Fig. 1c. While, the average thickness could be well controlled. We take an analysis based on various characteristics of films with different thicknesses to examine the reliability of nominal thickness. The spatial fluctuation of thickness will be discussed in SI F. Fig. S5 shows the magneto-transport measurement results of samples post-annealed for 30 min. with different thicknesses.

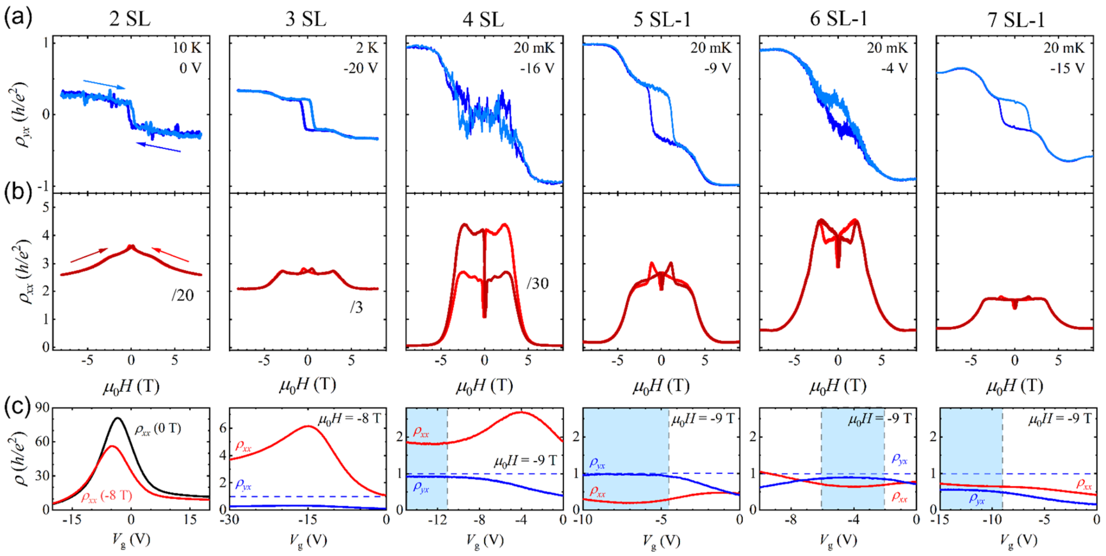

Figure S5. Magneto-transport measurements of samples post-annealed for 30 minutes with different thicknesses. (a, b) The ρ_{yx}–$\mu_0 H$, ρ_{xx}–$\mu_0 H$ curves with different thicknesses. (c) The corresponding anti-symmetrized ρ_{yx}–V_g and symmetrized ρ_{xx}–V_g curves of each sample at high field with the same temperature in (a). For the 2-SL sample, the ρ_{xx} (0 T)–V_g and ρ_{xx} (-8 T)–V_g curves are shown.

Temperature evolution of magneto-transport measurements in films with different thicknesses

The temperature evolution of magneto-transport measurements in typical 3-SL to 7-SL films with O_2 exposure are shown in Fig. S6. The ρ_{xx} (0 T)–T curves of all films exhibit a clear cusp around T_N (Fig. S6a). At CNP, ρ_{xx} always increase with decreasing temperature under zero field (Fig. S6c), in contrast to the metallic behavior in the edge state dominant regimes under high field (Fig. S6c. See more details below 3 K in Fig. S15). With elevated temperature approach to T_N, all samples show a weakened anomalous Hall effect at zero field and a shrunken coercive field (H_c) (Fig. S6b), suggesting the gradual destruction of long-range magnetic order. Meanwhile, all $|\rho_{yx}|$ (±9 T) decrease due to the emergence of dissipative channels, leading to the breakdown of quantization.

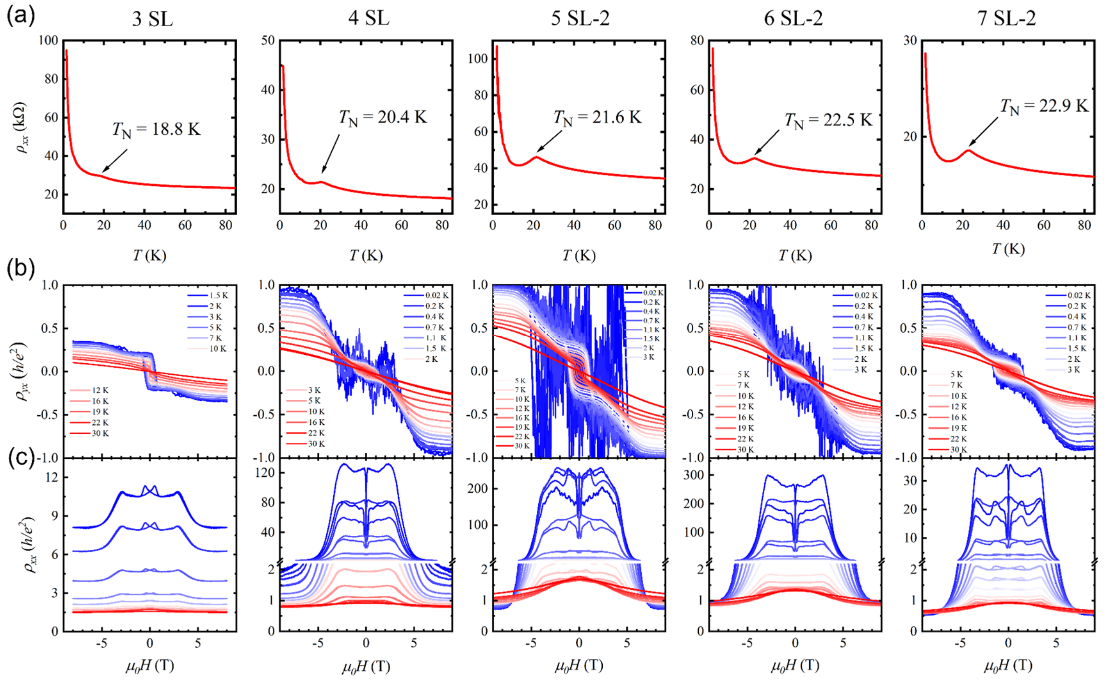

Figure S6. The temperature evolution of magneto-transport measurements in films with different thicknesses. 5-SL to 7-SL films were post-annealed for 120 min. (a) The ρ_{xx} (0 T)–T curves of films with different thicknesses at V_g = 0 V. (b, c) The temperature evolution of ρ_{yx}–μ_0H, ρ_{xx}–μ_0H curves at CNP with different thicknesses, respectively.

Thickness-dependent transport characteristics

To examine the controllability of thickness, the thickness-dependent T_N, $|\rho_{yx}|$ at 0 T (ρ_{AHE}) and H_c of films extracted from Fig. S6 are shown in Fig. S7. Both ρ_{AHE} and H_c tend to vanish when T is approaching to T_N, indicating the validity of extractive data (Figs. S7b and S7c). Firstly, T_N shows a positive correlation with the thickness due to the enhanced anisotropy energy and arrives at about 23 K in the 7-SL film, comparable to the bulk value (Fig. S7a) [11]. Thin films with same growth time have similar T_N, indicating the repeatability of nominal thickness. Secondly, ρ_{AHE} shows an obviously even-odd-SL oscillatory behavior dependent on different net magnetizations (Fig. S7b). While, the oscillation of H_c [11] is missing in our results (Fig. S7c). A possible explanation is that H_c depends on the spin configuration, which is sensitive to sample details and local environments. In MBE-grown films, as the topography shown in Fig. 1c, random islands and depressions (tens to hundreds of nanometers) within 1-SL thickness may have impact on the process of Zeeman energy competing against anisotropy energy and exchange energy. $|\rho_{yx}|$ at 0 T, on the other hand, is mainly dependent on the net magnetization, whose oscillatory behavior supports the average thickness is well controlled. These results could elucidate the reliability of nominal thickness, at least in a mean field view. It is also worth mentioning that the ρ_{AHE} as a function of nominal thickness (estimated by the growth time) could show a well even-odd-SL oscillatory behavior as expected without a shift on thickness, which indicates in-situ medium O_2 exposure has negligible effect on thickness.

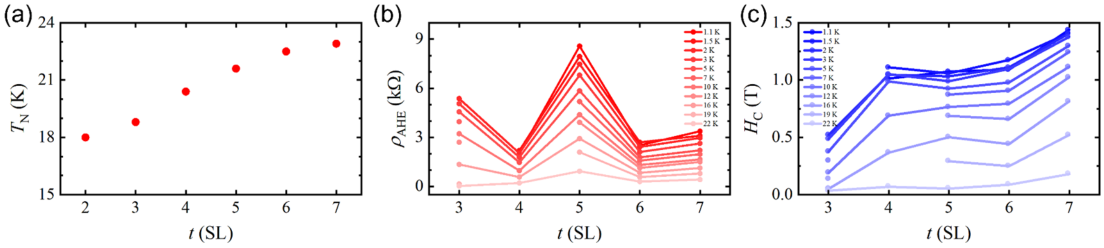

Figure S7. Various characteristics of films with different thicknesses. (a–c) T_N, ρ_{AHE} and H_c versus thickness, respectively.

SI D: High repeatability and scalability of MBE-grown films

High repeatability

To illustrate the high repeatability of MBE-grown films, transport results of more 5-SL samples are shown in Figs. S8 and S9, except the two 5-SL samples displayed in the main text. Since the measurements in a dilution refrigerator take a long time, most samples were calibrated at around 1.6 K. Magneto-transport measurement results of three 5-SL samples with 30 min. post-annealing are shown in Fig. S8. As for elongated post-annealed samples, sample 5 SL-2 has a ρ_{yx} (-9 T) of about 23.7 kΩ (0.92h/e^2) at 1.5 K (Fig. 3a), and sample 5 SL-6a with 300 min. post-annealing has a ρ_{yx} (-8 T) of 23.1 kΩ (0.89h/e^2) at 1.6 K (Fig. S9c), both of which are comparable and show nearly quantized Hall resistivity. The similar transport behaviors indicate the high repeatability of MBE-grown thin films.

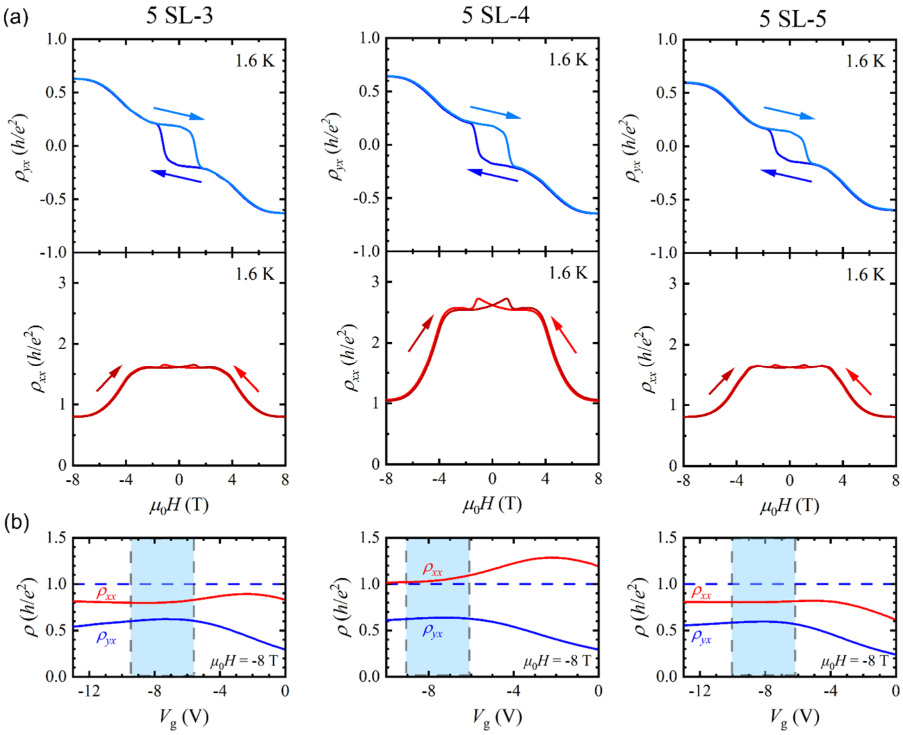

Figure S8. Magneto-transport measurements of other representative 5-SL samples with 30 min. post-annealing. (a) The ρ_{yx}–μ_0H and ρ_{xx}–μ_0H curves at CNP of three repeated samples measured at 1.6 K. (b) The ρ (-8 T)–V_g curve of each sample at 1.6 K, respectively.

Scalability

To elucidate the scalability and homogeneity of MBE-grown $MnBi_2Te_4$ thin films, two devices fabricated on different areas of the same film were measured. Limited by the facility, the maximum of film size is about 5 mm. One set of representative devices, 5 SL-6a and 5 SL-6b, were fabricated on the top and bottom areas of a cutting-off 5-SL film (roughly 3 mm × 5 mm), which was post-annealed for 5 hours under Te flux. The optical microscopy image of each device is shown in the insets of Fig. S9a. With individual measurements, two devices show quite similar transport behaviors at around 1.6 K, indicating the homogeneity of our macroscopic samples. This result reveals the good scalability of MBE-grown thin films.

In principle, Hall bar devices could be fabricated in millimeter scale by virtue of the homogeneity in MBE-grown films. However, to avoid a large leakage current, the Hall bar size of 40 μm × 80 μm is applied in this work, limited by the quality of dielectric layer AlO_x deposited at a low temperature (< 60°C), rather than that of films themselves.

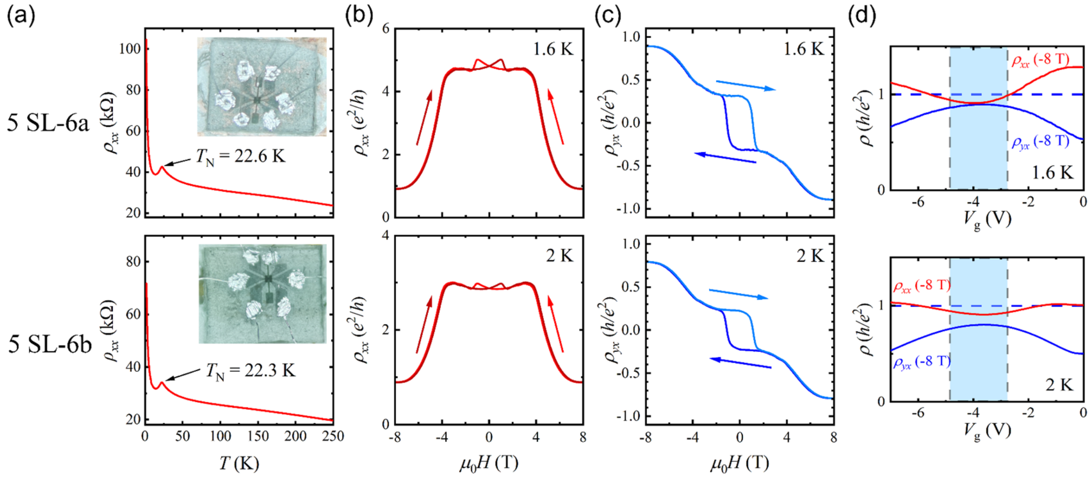

Figure S9. Homogeneity test of two devices on the same sample. Two devices (5 SL-6a, 5 SL-6b) were measured individually. (a) The ρ_{xx} (0 T)–T curves with $V_g = 0$. The optical microscopy images of two devices are shown in the insets, respectively. (b, c) The ρ_{xx}–μ_0H, ρ_{yx}–μ_0H curves at CNP measured at around 1.6 K, respectively. (d) The ρ (-8 T)–V_g curve of each sample measured at around 1.6 K.

SI E: More STEM cross-sectional images of MBE-grown films

We show more detailed STEM cross-sectional images of the 5-SL film with 30min. post-annealing in Fig. 1d. Certainly, the majority of sample has septuple-layer structures. While, rare defects can also be found in some regions, such as the typical two shown in Fig. S10. Guiding with the red dashed lines, boundaries of 180° rotational domains (Fig. S10a) and dislocations connecting the quintuple-layer and septuple-layer structures accompanied with bilayer structures (Fig. S10b) can be observed. These structural defects might be responsible for the inhomogeneity, leading to the finite residual ρ_{xx} in the Chern insulator state (Fig. 2c).

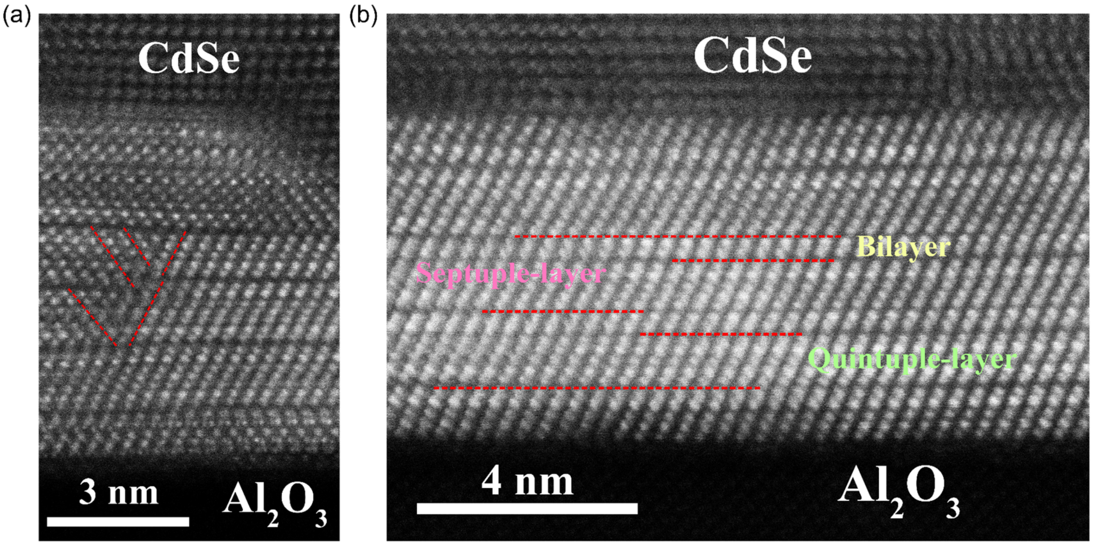

Figure S10. More STEM cross-sectional images of the 5-SL sample used in Fig. 1d. (a) Boundaries of 180° rotational domains. (b) Dislocations connecting different atomic structures. The red dashed lines are guide to eye.

SI F: Statistical results on different post-annealing time and coverages

In this section, we discuss the impacts on transport properties from elongated post-annealing and that on the ground state at zero field from spatial fluctuation of thickness.

5-SL films with different post-annealing time

More statistical results of samples used in Figs. 3c and 3d are shown in Fig. S11. The relations between ρ_{xx} (0 T) at 1.6 K and n, ρ_{yx} (0 T) at 1.6 K and n are shown in Figs. S11a and S11b, respectively. Here n is estimated from the ordinary Hall effect at 25 K and all data are extracted at CNP ($V_g = V_g^0$). Both relations have a similar tendency, a larger longitudinal (anomalous Hall) resistivity accompanied with a lower n. For ρ_{xx} (0 T), it can be easily understood with the Einstein relation $\sigma_{xx} = e^2 N_F D$, where N_F is the density of states at Fermi level and D is the diffusion coefficient. The mobility, which is related to D, has no significant variation in samples with different post-annealing time (Fig. S11c). A lower n at CNP in samples with elongated post-

annealing leads to a lower σ_{xx} at zero field. In the situation of $\sigma_{xx} >> \sigma_{xy}$, which is loosely satisfied in the zero-field case at 1.6 K (Figs. 4a and 4b), the relation between 2D resistivity and conductivity can be written as $\rho_{xx} \approx 1/\sigma_{xx}$, $\rho_{yx} \approx \sigma_{xy}/\sigma_{xx}^2$. Thus, a lower σ_{xx} (0 T) corresponds to a larger ρ_{xx} (0 T). As for ρ_{yx} (0 T), it is not surprising since anomalous Hall resistivity usually has a positive correlation with longitudinal resistivity whatever from intrinsic or extrinsic contribution [12]. As for the residual ρ_{xx} in the FM configuration, it has been discussed in details in the main text. An elongated post-annealing leads to a higher quality of thin films with less chemical potential fluctuations.

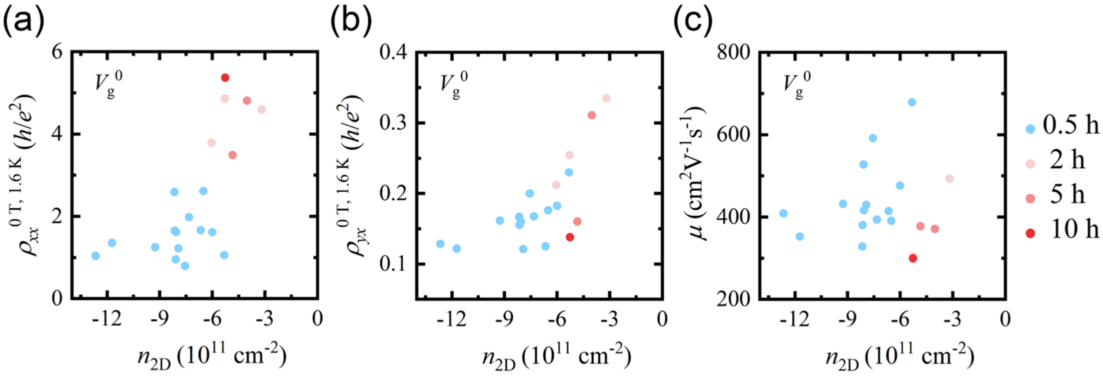

Figure S11. Statistical results of 5-SL samples with different post-annealing time. (a, b) The relations between ρ_{xx} (0 T) at 1.6 K and n, ρ_{yx} (0 T) at 1.6 K and n, respectively. (c) The relation between mobility μ (25 K) and n. The data of each sample is taken at CNP. Different colors represent different post-annealing time.

To further elucidate the scenario of QAH puddles coupled with each other in elongated post-annealed samples, a preliminary test has been done. Two devices with different sizes (one is 30 μm × 30 μm and the other is 40 μm × 80 μm) were fabricated on a 5-SL film with 120 min. post-annealing. As the magneto-transport results at 1.6 K shown in Fig. S12, transport behaviors and the magnitude of ρ_{yx} (-8 T) are similar in two samples, indicating the comparable sample quality. While, the magnitude of ρ_{xx} is significantly different, i.e. a smaller ρ_{xx} (0 T) and ρ_{xx} (-8 T) exist in the sample with a smaller size. The change in ρ_{xx} (0 T) can be understood with the localization effect, which is related to the sample size and localization length. The smaller ρ_{xx} (-8 T) might support the picture that the sample is composed of QAH puddles, since fewer puddles could exist between longitudinal electrodes in a smaller device leading to fewer tunneling processes. A systematic study on different device sizes would be helpful to clarify this in the future.

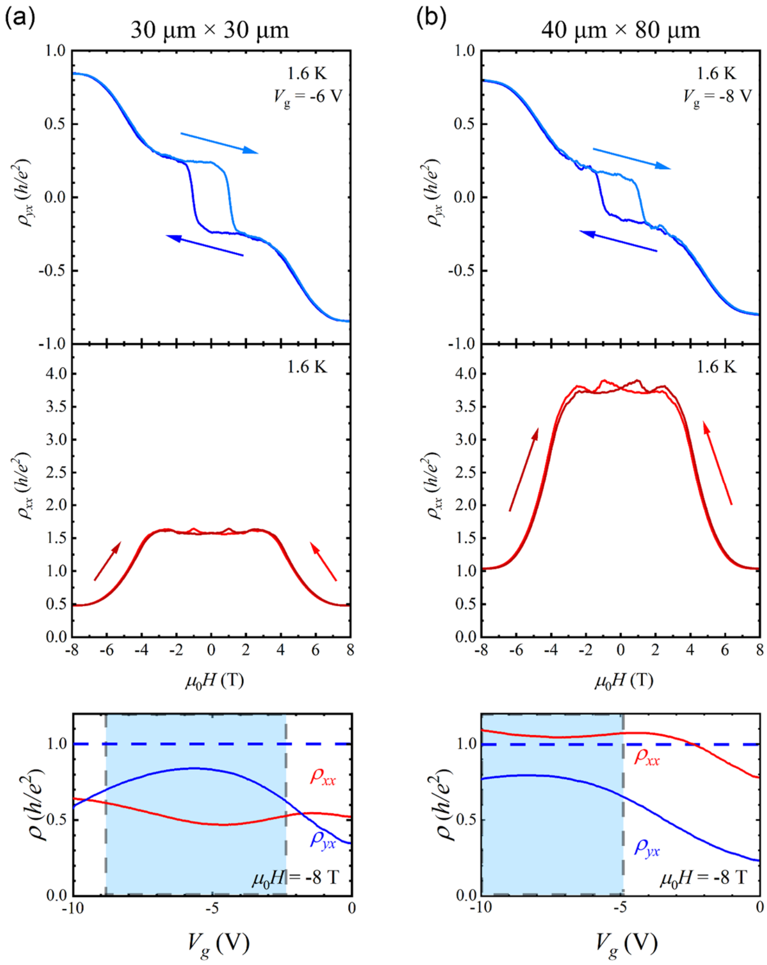

Figure S12. Magneto-transport measurements of an elongated post-annealed film with different device sizes. (a, b) The ρ_{yx}–μ_0H and ρ_{xx}–μ_0H curves at CNP and ρ (-8 T)–V_g curves of two devices with the Hall bar size of 30 μm × 30 μm, 40 μm × 80 μm, respectively. The measurements were performed at 1.6 K.

5-SL films with different coverages of even-SL region

Next we discuss about the potential impacts on the ground state at zero field from spatial fluctuation of thickness. Theoretical calculations predict that the QAH state and axion insulator state are the ground states of odd- and even-SL $MnBi_2Te_4$ films at zero field, respectively. Chiral edge states would emerge at the step edges of different layers, patterned by the topography of samples. In this picture, our thin films could be considered as a mixture of QAH state regions and axion insulator state regions. In previous theoretical and experimental works, a quantum phase transition could occur accompanied with $\rho_{xx} \sim 1h/e^2$ by tuning the proportion of axion insulator state and QAH state (about 50% of each state) in magnetically modulation-doped TI films [13,14]. Different ratio of two states has a drastic impact on ρ_{xx}, that is to say, extremely large ρ_{xx} with an insulating behavior when the axion insulator state is dominant and small ρ_{xx} with a metallic behavior when the QAH state is dominant. It can be understood since the macroscopic system would show properties of the dominant state governed by the quantum percolation [15]. In our case, the proportion of different states could be estimated by the coverage. As shown in Fig. S13, the total coverage of

4-SL and 6-SL regions ($\delta_{4\ \mathrm{SL}\ +\ 6\ \mathrm{SL}}$), in each 5-SL sample shown in Figs. S11 is in the range of 20% to 45%. No significant correlation of ρ_{xx} (0 T) at 1.6 K with the coverage of even-SL regions has been observed (Fig. S13b). While, a larger ρ_{xx} (0 T) in samples with elongated post-annealing can be easily seen, the reason of which is discussed above. These results indicate that, to some extent, ρ_{xx} (0 T) might not be very sensitive to the inhomogeneous thickness in our films. Therefore, the insulating behavior of sample 5 SL-1 (well quantized in the FM state as shown in Fig. 2c) at zero field, $\delta_{4\ \mathrm{SL}\ +\ 6\ \mathrm{SL}}$ of which is nearly 20%, might cannot be illustrated by the fluctuation of thickness simply. The insulating behaviors of our 5-SL films at zero field indicate their ground state deviates from the QAH state.

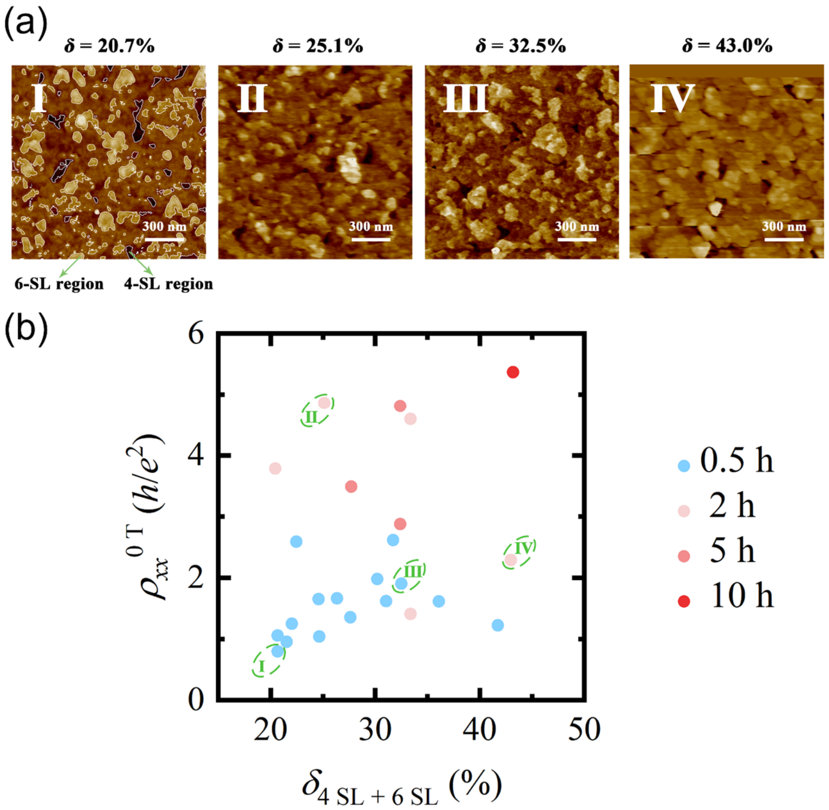

Figure S13. Statistical results of 5-SL samples with different coverages of 4-SL and 6-SL regions. (a) The topographies of four typical samples [as labeled in (b)] with different $\delta_{4\ \mathrm{SL}\ +\ 6\ \mathrm{SL}}$. An example is shown in the first figure (I) to illustrate the estimated even-SL regions (surrounded by white lines). The size of each scanning region is 1.5 μm × 1.5 μm. (b) The relation between ρ_{xx} (0 T) at 1.6 K and $\delta_{4\ \mathrm{SL}\ +\ 6\ \mathrm{SL}}$. The data of each sample is taken at CNP. Different colors represent different post-annealing time.

More comparisons between films with different post-annealing time

Similar to 5-SL films, the elongated post-annealing effect, a better quantized ρ_{yx} accompanied by a larger ρ_{xx} in the FM configuration and a significantly larger ρ_{xx} at zero field, is also observed in 6-SL and 7-SL films (Fig. S14a).

Take the comparison between samples 5 SL-1 and 5 SL-2 as an example, temperature dependences of σ_{xx} in the Chern insulator state between samples with different post-annealing time is different. As shown in Fig. S14b, when $T < 2$ K ($T^{-1} >$

0.5 K^{-1}), σ_{xx} (-8 T) of sample 5 SL-1 varies fast with temperature, while, σ_{xx} (-8 T) of sample 5 SL-2 seems to be insensitive. As we discussed in the main text, the residual resistivity of sample 5 SL-1 comes from dissipative bulk channels (Fig. 3f), which is sensitive to temperature because of thermal activation. While, ρ_{xx} (-8 T) of sample 5 SL-2 might origin from the tunneling between QAH puddles. The tunneling process mainly relies on the potential of saddle point and correlation length, which might be not too sensitive to T. The different temperature dependences of residual longitudinal conductivity indicate their different origins of dissipation. At zero field, ρ_{xx} of sample 5 SL-2 has a dramatic variation compared with sample 5 SL-1, about two orders of magnitude larger at 20 mK (Fig. S14c).

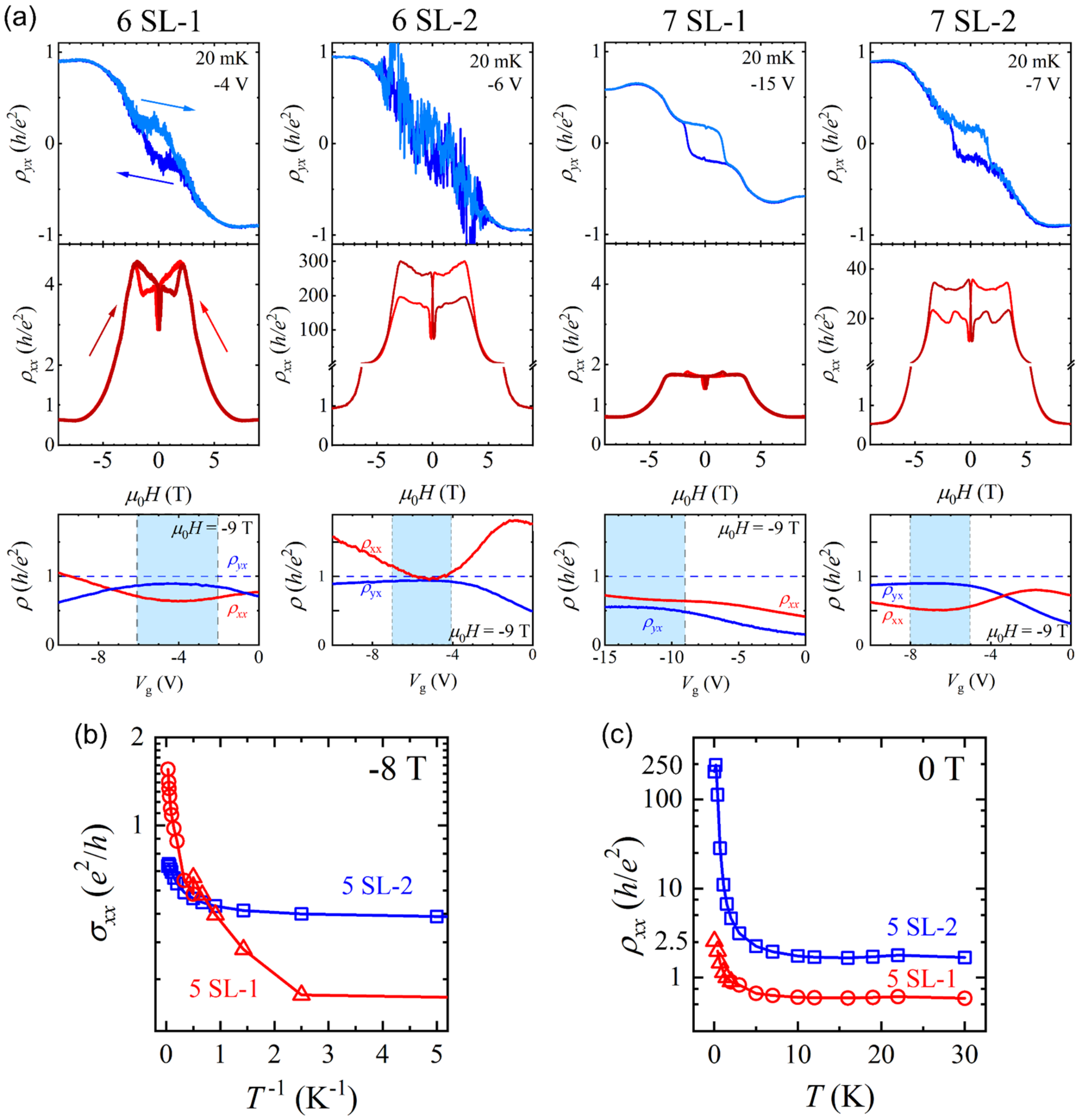

Figure S14. More comparisons of samples with different post-annealing time. (a) The ρ_{yx}–μ_0H, ρ_{xx}–μ_0H and ρ (-9 T)–V_g curves of samples with 30 min. post-annealing (6 SL-1, 7 SL-1) and samples with 120 min. post-annealing (6 SL-2, 7 SL-2) at 20 mK, respectively. The data of samples 6 SL-1 and 7 SL-1 is redrawn from Fig. S5 for convenient comparison. (b) Logarithmic-scale plot of σ_{xx}–T^{-1} curves of samples 5 SL-1 and 5 SL-2 at -8 T. (c) Logarithmic-scale plot of ρ_{xx}–T curves of samples 5 SL-1 and 5 SL-2 at 0 T. Different symbols of sample 5 SL-1 in (b) and (c) represent data taken from different transport measurement systems.

SI G: Quantum phase transitions in films with different thicknesses

Due to different ground states existing at zero field and high field in our samples, it is naturally to expect a quantum phase transition occurring with magnetic field. Fig. S15 shows ρ_{xx} (μ_0H, T) of films with different thicknesses near their critical fields (H_{cr}s) below 3 K. In most samples, the quantum phase transitions have occurred with ρ_{cr} close to h/e^2 (Fig S15). It meets the theoretical prediction well as the critical point of quantum phase transition is suggested near ($0.5e^2/h$, $0.5e^2/h$) in σ space [16]. The metal to insulator transitions are absent in samples 4 SL and 6 SL-2. One possible explanation might be that more insulating regions in such two samples hinder the percolation of QAH edge states [15], indicated by a slightly larger residual ρ_{xx} (9 T) of them. While, the tendencies of ρ_{xx} (μ_0H, T) in such two samples seem to be crossing at a larger magnetic field, suggesting that a better magnetization may facilitate the transition. In sample 7 SL-1, the crossing point of ρ_{xx}-μ_0H at different temperatures cannot be well defined. A simple reason might be that the sample has not been in the QAH state completely under high field, still accompanied with residual bulk channels. It's worth mentioning that the universality statement concerns the exponent value κ (insets of Fig. S15) while not showing the requirement on H_{cr}, which is reliant on the microscopic details [17]. Thus, the difference of H_{cr} between our samples and exfoliated thin flakes is not surprising.

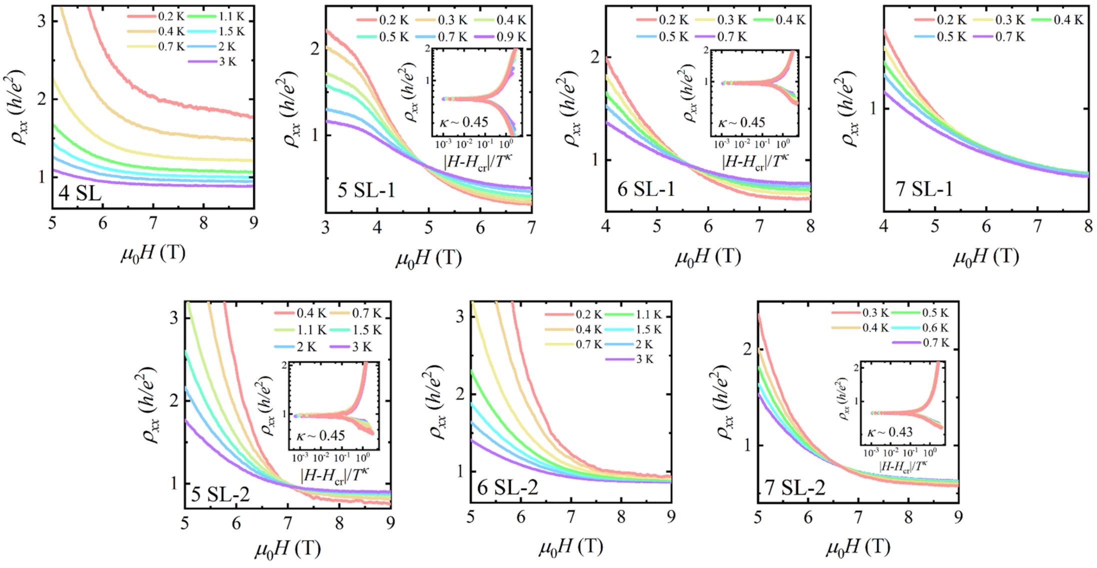

Figure S15. Quantum phase transitions in films with different thicknesses. ρ_{xx} (μ_0H, T) of different samples near their H_{cr}s below 3 K. Samples 4 SL, 5 SL-1, 6 SL-1 and 7 SL-1 were post-annealed for 30 minutes. Samples 5 SL-2, 6 SL-2 and 7 SL-2 were post-annealed for 120 minutes. The data of each sample is taken at CNP. The insets show the scaling analyses. The data of samples 5 SL-1 and 5 SL-2 is redrawn from Figs. 4d and 4e for convenient comparison.

References

1. Chen YL, Chu JH, Analytis JG *et al.* Massive Dirac fermion on the surface of a magnetically doped topological insulator. *Science* 2010; **329**: 659–62.
2. Du MH, Yan J, Cooper VR *et al.* Tuning Fermi levels in intrinsic antiferromagnetic topological insulators $MnBi_2Te_4$ and $MnBi_4Te_7$ by defect engineering and chemical doping. *Adv Funct Mater* 2021; **31**: 2006516.
3. Yan JQ, Okamoto S, McGuire MA *et al.* Evolution of structural, magnetic, and transport properties in $MnBi_{2-x}Sb_xTe_4$. *Phys Rev B* 2019; **100**: 104409.
4. Chen B, Fei F, Zhang D *et al.* Intrinsic magnetic topological insulator phases in the Sb doped $MnBi_2Te_4$ bulks and thin flakes. *Nat Commun* 2019; **10**: 4469.
5. Koleini M, Frauenheim T and Yan B. Gas doping on the topological insulator Bi_2Se_3 surface. *Phys Rev Lett* 2013; **110**: 016403.
6. Hou F, Yao Q, Zhou CS *et al.* Te-vacancy-induced surface collapse and reconstruction in antiferromagnetic topological insulator $MnBi_2Te_4$. *ACS Nano* 2020; **14**: 11262–72.
7. Li H, Liu S, Liu C *et al.* Antiferromagnetic topological insulator $MnBi_2Te_4$: synthesis and magnetic properties. *Phys Chem Chem Phys* 2020; **22**: 556–63.
8. Akhgar G, Li Q, Bernardo ID *et al.* Formation of a stable surface oxide in $MnBi_2Te_4$ thin films. *ACS Appl Mater Interfaces* 2022; **14**: 6102–8.
9. Mazza AR, Lapano J, Meyer III HM *et al.* Surface-driven evolution of the anomalous Hall effect in magnetic topological insulator $MnBi_2Te_4$ thin films. *Adv Funct Mater* 2022; **32**: 2202234.
10. Fan Y, Kou X, Upadhyaya P *et al.* Electric-field control of spin–orbit torque in a magnetically doped topological insulator. *Nat Nanotech* 2016; **11**: 352–9.
11. Yang S, Xu X, Zhu Y *et al.* Odd-even layer-number effect and layer-dependent magnetic phase diagrams in $MnBi_2Te_4$. *Phys Rev X* 2021; **11**: 011003.
12. Nagaosa N, Sinova J, Onoda S *et al.* Anomalous Hall effect. *Rev Mod Phys* 2010; **82**: 1539–92.
13. Xiao D, Jiang J, Shin JH *et al.* Realization of the axion insulator state in quantum anomalous Hall sandwich heterostructures. *Phys Rev Lett* 2018; **120**: 056801.
14. Wu X, Xiao D, Chen CZ *et al.* Scaling behavior of the quantum phase transition from a quantum-anomalous-Hall insulator to an axion insulator. *Nat Commun* 2020; **11**: 4532.
15. Lee DH, Wang Z and Kivelson S. Quantum percolation and plateau transitions in the quantum Hall effect. *Phys Rev Lett* 1993; **70**: 4130–3.
16. Burgess CP, Dib R and Dolan BP. Derivation of the semicircle law from the law of corresponding states. *Phys Rev B* 2000; **62**: 15359–62.
17. Pruisken AMM. Universal singularities in the integral quantum Hall effect. *Phys Rev Lett* 1988; **61**: 1297–300.
